# Laying the Foundation for Pregnancy Physical Activity Profiling: A Framework for Providing Tailored Physical Activity Advice and Guidance to Pregnant Women

**DOI:** 10.3390/ijerph18115996

**Published:** 2021-06-03

**Authors:** Marlize De Vivo, Hayley Mills

**Affiliations:** School of Psychology and Life Sciences, Faculty of Science, Engineering and Social Sciences, Canterbury Christ Church University, Kent CT1 1QU, UK; hayley.mills@canterbury.ac.uk

**Keywords:** physical activity, exercise, pregnancy, theory of planned behaviour, intention, behaviour, past behaviour, profiling

## Abstract

The aim of this study was to examine the predictive utility of the theory of planned behaviour (TPB) in explaining pregnant women’s physical activity (PA) intentions and behaviour and to scrutinise the role of past behaviour within this context. Pregnant women (*n* = 89) completed the pregnancy physical activity questionnaire (PPAQ) and newly developed TPB questionnaire on two separate occasions during their pregnancy. Analyses were carried out in relation to three scenarios. Firstly, when considering the original TPB, intention emerged as the strongest determinant of pregnant women’s PA behaviour. Secondly, controlling for past behaviour attenuated the influence of intention and perceived behavioural control on behaviour, with neither of the original variables providing a unique influence. Finally, the addition of past behaviour added significantly to the prediction of intention with the model as a whole, explaining 85% of the variance in pregnant women’s PA intention, and with past behaviour uniquely contributing 44.8% of the variance. Pregnancy physical activity profiling based on intention and behaviour status is subsequently introduced as a novel and practical framework. This provides healthcare professionals with the opportunity and structure to provide tailored advice and guidance to pregnant women, thereby facilitating engagement with PA throughout motherhood.

## 1. Introduction

Whilst pregnancy is consistently associated with a decline in physical activity (PA) levels [1,2], it is increasingly recognised that pregnant women’s lifestyle choices can impact the health of the mother and baby and that these effects can be observed beyond gestation and birth [3]. For example, regular PA during pregnancy contributes to a reduction in hypertensive disorders, improved cardiorespiratory fitness, lower gestational weight gain and a reduction in risk of gestational diabetes [4]. In acknowledgement of the mounting evidence supporting these benefits and the accompanying responsibility to encourage regular PA in the female population, it is necessary to draw on relevant theories of behaviour and/or behaviour change to fully understand the nature of the modifiable factors involved, develop appropriate behaviour change interventions, and improve professional practice [5,6,7].

Despite the abundance of theories representing sets of ideas which aim to explain phenomena, Ayers and Olander [8] maintain that research involving pregnancy behaviours remains predominantly atheoretical. Indeed, a recent systematic review identified only 11 independent studies utilising theory in the examination of PA behaviour during pregnancy [9]. One theoretical framework that can be viewed as a starting point for understanding PA behaviour during pregnancy and one which is flexible enough to consider the role of past behaviour is Ajzen’s theory of planned behaviour (TPB) [10]. Indeed, it is regarded by the scientific community as a foundation from which revisions, expansions and new theories can progress [11]. This viewpoint is justified by the fact that the TPB involves “major theoretical constructs that have proved their utility over the years” and in various forms and combinations constitute the basis for several theories of behaviour and behaviour change [12] (p. 401). Thus, whilst it is acknowledged that PA behaviour may ultimately be best represented by a hybrid theory, the TPB provides a vantage point from which these developments can follow.

In their earlier work concerning the theory of reasoned action (TRA), Ajzen and Fishbein [13] maintained that behaviour can be explained in terms of a limited number of concepts; specifically, an individual’s ultimate behaviour is influenced primarily by their intention to perform (or not perform) that behaviour. Intention, in turn, is a function of two determinants, one personal in nature (i.e., attitude) and the other representing social influence (i.e., subjective norm) [13]. Attitude in this context is defined as a disposition to respond with some degree of favourability or unfavorability to a certain behaviour or object [12]. Thus, an individual who believes that engaging in a certain behaviour will result in mostly positive consequences will hold a favourable attitude toward performing that behaviour, whereas an individual who believes that performing that behaviour will lead to mostly negative consequences will hold an unfavourable attitude [13]. Subjective norm refers to the perceived social pressure to engage in a specific behaviour; therefore, an individual will perceive social pressure to engage in a certain behaviour if they believe that most people whose opinion they value think that they should perform that behaviour, whereas an individual will perceive social pressure to avoid a certain behaviour if they believe that most people whose opinion they value think that they should not perform that behaviour [12,13].

The TRA was, however, only concerned with behaviours under complete volitional control, and in order to account for behaviours where control was insufficient, a third determinant, perceived behavioural control (PBC), was added by Ajzen [10]. PBC refers to an individual’s perception of their ability to perform a certain behaviour and whether they have control over its performance [13]. This extended theory, the TPB, has since been used to predict and explain a variety of behaviours, including smoking cessation [14], breastfeeding [15], binge drinking [16], blood donation [17], screening uptake [18] and exercise [19]. Indeed, several reviews and meta-analyses have been produced attesting to its effectiveness and illuminating the relationships between constructs in different contexts (see [20,21,22,23,24]).

In recent years, the TPB has, however, come under the spotlight, with its utility questioned by Sniehotta, Presseau, and Araújo-Soares [25]. Renewed interest, certainly from Ajzen [26] himself, was seen in response to address some of the issues raised (for commentaries, see [27,28,29,30,31,32,33,34,35]). What became clear is that very few researchers made the effort to carry out formative research to inform both TPB studies and behaviour change interventions, an approach that Ajzen [26] considers “cavalier” (p. 4) indicating “a profound misunderstanding of the theory itself” (p. 6). Through a recent three-level meta-analysis spanning across domains, Steinmetz, Knappstein, Ajzen, Schmidt, and Kabst [36] showed that interventions based on the TPB were effective in changing behaviours [δ = 0.50 (95% CI: 0.24–0.75)]. They also found that increasing skills, persuasion and motivation were successful behaviour change methods associated with TPB interventions. These findings certainly rebut Sniehotta and colleagues’ [25] comments regarding the inability of the TPB to assist researchers and practitioners in developing appropriate interventions.

One acknowledged strength of the TPB is that it is considered a flexible framework that allows the incorporation of other variables, for example, the integration of past behaviour as an additional predictor variable has been consistently reported to account for a further variance on intentions of approximately 10% [15]. In their meta-analysis, Hagger et al. [11] showed that while past behaviour weakened relationships between TPB constructs, it did not eliminate the effects of attitudes on intentions, intentions on behaviour, or PBC on behaviour. Past behaviour, however, emerged as a significant predictor of future behaviour (β = 0.55, *p* < 0.01), intention (β = 0.37, *p* < 0.01), attitude (β = 0.39, *p* < 0.01), subjective norm (β = 0.05, *p* < 0.01), PBC (β = 0.23, *p* < 0.01), and self-efficacy (β = 0.45, *p* < 0.01). Furthermore, McEachan, Conner, Taylor, and Lawton’s [37] meta-analysis pertaining to health behaviours (including risk, detection, PA, dietary, safe sex, and abstinence behaviours) reported the inclusion of past behaviour to contribute an additional 10.9% variance to the prediction of behaviour and 5% to the variance in intention. Past behaviour also emerged as the strongest predictor of future behaviour but not intention.

In attempting to understand the role of past behaviour, researchers often reason that frequent performance of a behaviour in the past leads to the formation of habits or behavioural tendencies which are than more likely to result in automatic responses [38]. The “automatic habit hypothesis” implies that intention progressively becomes irrelevant as habits are formed [12] (p. 52). Thus, in stable contexts, it is argued that future behaviour will be influenced directly by past behaviour; however, when habits are unlikely to develop due to lack of opportunity to engage regularly in a specific behaviour or when a change in context occurs, the effect of past behaviour is said to indirectly influence future behaviour through consciously formed intentions [38]. However, neither the meta-analysis nor the original study carried out by Ouellette and Wood [38] could support this hypothesis.

With regards to pregnancy, De Vivo and colleagues [39] point out that only two studies have considered the effects of past behaviour using the TPB but did so in different ways. Zamora-Flyr [40] reported that walking behaviour during the second trimester predicted walking behaviour during the third trimester; that is, PA behaviour could be predicted within the context of pregnancy. Contrastingly, Hausenblas, Symons Downs, Giacobbi, Tuccitto and Cook [41] did not find preconception PA behaviour to predict pregnancy PA behaviour; that is, PA behaviour outside the context of pregnancy did not predict PA within the context of pregnancy. Yet, in their systematic review of behaviour change interventions during pregnancy, Currie and colleagues [42] report that “PA at baseline has the potential to influence the outcome to a greater extent than the intervention itself” (p. 11). This is important as PA levels decrease as pregnancy progresses and do not usually return to preconception levels [2], indicating that inactivity at baseline may set women up for continued inactivity throughout motherhood.

The aim of this study is therefore to examine the predictive utility of the TPB in explaining pregnant women’s PA intentions and behaviour and to scrutinise the role of past behaviour within the context of pregnancy. Specifically, it is hypothesized that: (1) concerning the original TPB, intention will be the strongest determinant of PA behaviour, and attitude will be the strongest determinant of intention; (2) when controlling for past behaviour, the influence of intention and PBC on future behaviour will be attenuated with unique influences remaining, and the influence of attitudes, subjective norms and PBC on intentions will be attenuated with unique influences remaining; and (3) when adding past behaviour as additional variable, the model will explain significantly more variance in pregnant women’s PA intentions and behaviour.

## 2. Methods

### 2.1. Procedures

This study forms part of a larger multiphase research project with the aim of predicting and understanding PA behaviour during pregnancy. Ethical approval to conduct the research was granted by the National Research Ethics Service (NRES) Committee of London—Camberwell St. Giles (reference number: 13/LO/139) and permission to carry out the study was provided by the East Kent Hospitals University NHS Foundation Trust (EKHUFT; reference number: 2013/WOMHE/01).

In line with best practice and due to the contextual sensitivity of TPB studies [12,43,44], an elicitation study was conducted to identify pregnant women’s behavioural, normative and control beliefs in relation to taking part in physical activities. These beliefs were subsequently used to construct a tailored questionnaire to measure the TPB constructs in a relation to the pregnant women’s PA behaviour.

Consistent with the procedure recommended by Fishbein and Ajzen [12], a small sample of pregnant women (*N* = 18) were recruited and asked to complete a questionnaire using open-ended questions to describe their beliefs about taking part in PA. A content analysis of the pregnant women’s beliefs was performed by grouping responses into themes and subsequent labelling of categories with suitable tags [44]. Content validity was established by having the second author check the analysis process and categorization [45]. In case of disagreement, grouping possibilities were considered until consensus was obtained. The strategy used to compile the modal set of beliefs is that suggested by Fishbein and Ajzen [12], whereby beliefs are selected based on their frequency of emission until 75% of all responses listed are accounted for (see Table 1, Table 2, Table 3 and Table 4).

Following content analysis, a draft version of the TPB questionnaire was developed. This questionnaire consisted of 53 items and contained both belief indices and direct measures of the TPB constructs. Of the 18 pregnant women who completed the beliefs questionnaire, seven piloted and provided feedback on the first draft of the TPB questionnaire. Following consideration of comments, minor amendments were applied before the questionnaire was implemented during the main study.

During the next phase, participants were recruited over a period of five months in 2015 when attending an appointment at one of ten randomly selected NHS antenatal clinics in East Kent. Pregnant women were eligible for inclusion if they were at least 18 years of age, proficient in the English language, had conceived naturally, had not had more than one previous miscarriage, had no previous or existing condition which might be caused or aggravated by pregnancy (e.g., asthma, diabetes, high blood pressure, etc.; [46,47]), and had not participated in a previous phase of the research project. Those who agreed to participate were asked to complete the consent form and demographics questionnaire on the same day and were then offered the choice of completing the main study questionnaires in written (paper) or electronic (online) format. Participants were subsequently required to complete study questionnaires on two separate occasions during their pregnancy.

### 2.2. Participants

**Figure 1:**
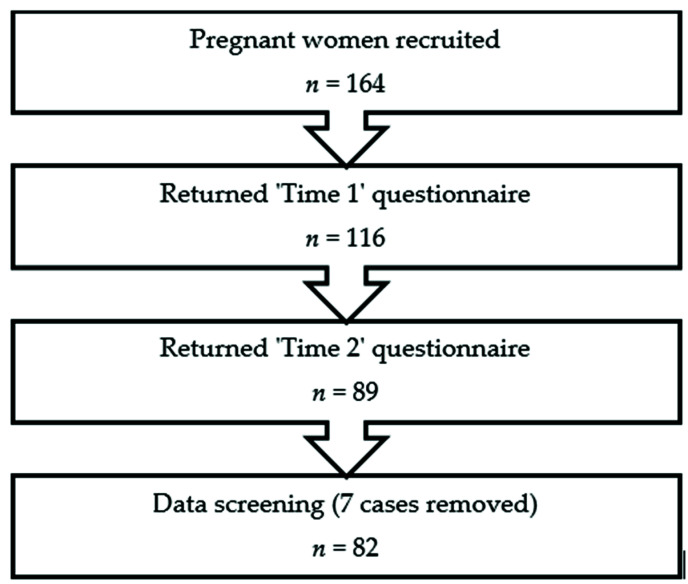
Participant flow.

Of the 164 pregnant women who consented to participate, 116 returned their “time 1” questionnaires and 89 completed the study. The average time between the completion of “time 1” and “time 2” questionnaires was three weeks (20.9 days). Non-responders at both “time 1” and “time 2” were sent three reminders before being excluded from the study. Following initial screening of data, seven cases were removed from the study: two sets of questionnaires had missing or incomplete data, three participants completed the questionnaire incorrectly, one data set displayed ‘response set’ answers, and one participant revealed that she was expecting twins and, therefore, did not meet the inclusion criteria (see Figure 1).

The majority of the remaining 82 participants described themselves as being of white ethnicity (93.90%) and having English (64.63%) or British (23.17%) nationality. Participants had an average age of 29.25 years (SD = 4.88), with most women being married (56.10%) and in full- or part-time employment (74.39%). Most participants (63.41%) reported having attained a level 4 education (i.e., certificate of higher education) or above. The average annual household income (i.e., representing the total income from all people living in the same household) as reported by 66 of the participants (80.49%) was £41,560.61. Half of the participants were recruited during their second trimester of pregnancy (weeks 13 to 28) with the average gestational age being 25.15 weeks (SD = 8.04). For 35 (42.68%) of the participants, this was their first pregnancy. Nearly two thirds of the participants (65.85%) reported taking part in PA on a regular basis in the 12 months prior to their pregnancy, whilst just over half (53.66%) of the participants reported that they were regularly active during their current pregnancy.

### 2.3. Measures

#### 2.3.1. Pregnancy Physical Activity Questionnaire (PPAQ)

The PPAQ is a self-administered instrument measuring the type, duration, frequency, and intensity of total activity in pregnant women [48]. This questionnaire has been found to be a reasonably accurate and reliable measure of PA in pregnant women with intra-class correlation coefficients (r) ranging from 0.78 to 0.93. Respondents were asked to report the amount of time spent engaging in 32 activities which comprise the following categories: household/caregiving (*n* = 13), occupational (*n* = 5), transportation (*n* = 3), sports and exercise (*n* = 8), and inactivity (*n* = 3). Participants also had the opportunity to report activities not captured by the questionnaire. The self-reported time spent per activity was subsequently multiplied by its corresponding intensity to determine a measure of average weekly energy expenditure (MET-hours per week). The compendium of physical activities [49] was used as a guide to identify metabolic equivalents (METs or intensity) for additional activities identified by participants. The average number of MET-hours per week performed in each activity category (e.g., occupational) were calculated. Activities were classified into sedentary (<1.5 METs), light (1.5 to <3.0 METs), moderate (3.0 to 6.0 METs) or vigorous (>6.0 METs) intensities. Finally, light, moderate and vigorous activities were summed to compute the average MET hours per week representing a respondent’s total activity. The PPAQ was completed on two separate occasions; “time 1” serving as a measure of total PA levels at baseline and questions were therefore opened with “since becoming pregnant…” whilst the “time 2” questionnaire established current total PA levels and questions were opened with “during the previous two weeks of your pregnancy…”.

#### 2.3.2. TPB Questionnaire

The guidelines provided by Ajzen [43], Fishbein and Ajzen [12], and Francis et al. [44] were used to inform the development of the TPB questionnaire.

In line with the guidelines for PA during pregnancy at the time of the study [47], participants were asked to rate each of the questionnaire items with regards to them taking part in moderate PA for 15 to 30 min on at least four days of the week during their pregnancy. To ensure compatibility with respect to the target, action, context, and time (TACT) elements of the behaviour under investigation, participants read the following statement prior to completing the questionnaire:

*In this section, we are interested in your opinion about taking part in regular moderate PA during your pregnancy. It is important to recognise that PA forms part of our daily lives and can take on many forms. Sometimes, we may not even realise that we are in fact exercising. For the purpose of this questionnaire, regular PA is defined as any moderate PA (e.g., yoga, gardening, water aerobics, housework, etc.) that requires you to expend energy whilst still being able to hold a conversation (e.g., walking briskly at a pace of three miles per hour) and is performed continuously for 15 to 30 min on at least four days of the week during your pregnancy.*

##### Behavioural Criterion

PA behaviour was assessed on two occasions using a behaviour statement involving both a dichotomous and frequency criterion.

The following items were used at “time 1” to establish “past behaviour”:

1. Have you been exercising regularly during your pregnancy? [dichotomous criterion]
2. So far during my pregnancy, I have exercised on ______ days of the week. [frequency criterion]

The following items were used at “time 2” to establish “behaviour”:

1. During the previous two weeks of your pregnancy, have you been exercising regularly? [dichotomous criterion]
2. During the previous two weeks of my pregnancy, I have exercised on ______ days of the week. [frequency criterion]

##### Behavioural Intentions

To achieve scale correspondence with PA behaviour, participants were asked to indicate the number of days of the week that they intended to engage in PA during their pregnancy. A single item to measure “intention performance” is consistent with previous research [50,51,52].

##### Attitude

Direct Measure of Attitude

The direct measurement of attitude involved the use of seven bipolar adjectives which were evaluative in nature (good/bad, useless/useful, foolish/wise, harmful/beneficial, pleasant/unpleasant, boring/exciting, unenjoyable/enjoyable). In line with Ajzen’s [43] recommendations, both instrumental and experiential items were included in the questionnaire and positive and negative endpoints were counterbalanced to reduce the possibility of response sets. The internal consistency score for the seven items was excellent (α = 0.93). The mean of the item scores represented an overall attitude score.

Behavioural Beliefs

The modal salient behavioural beliefs identified during the elicitation study (see Table 1) were converted into a set of statements representing the behavioural beliefs of pregnant women in East Kent. The “behavioural belief strength” statements consisted of five items and participants were asked to rate each item using a seven-point Likert scale ranging from 1 (extremely likely) to 7 (extremely unlikely). For example, “exercising regularly during my pregnancy will improve my fitness”.

“Outcome evaluations” (i.e., evaluation of the “behavioural belief strength” statement) were assessed in the form of an incomplete declaration with the response provided on a seven-point Likert scale ranging from 1 (extremely undesirable) to 7 (extremely desirable). For example, “improving my fitness during this pregnancy is…”

Each “behavioural belief strength” score was multiplied by the relevant “outcome evaluation” score and the resulting products summed across all the items to create a total behavioural beliefs score.

##### Subjective Norm

Direct Measure of Subjective Norm

The direct measurement of subjective norm involved both descriptive and injunctive norm items. Participants were asked to rate five items on a seven-point Likert scale ranging from 1 (strongly disagree) to 7 (strongly agree). The mean of the item scores represented an overall subjective norm score. The three items representing injunctive norms had a good level of internal consistency (α = 0.82 [53]). The higher-order construct of subjective norm consisted of four items with an acceptable internal consistency (α = 0.74 [53]). This finding is not surprising as it is recognized that injunctive and descriptive norms reflect different aspects of perceived social pressure and can therefore either echo each other or be contradictory [12].

Normative Beliefs

The modal salient injunctive and descriptive beliefs identified during the elicitation study (see Table 2 and Table 3) were converted into a set of statements representing the normative beliefs of pregnant women in East Kent. Normative beliefs were assessed with six items and participants were asked to rate each item using a seven-point Likert scale ranging from 1 (strongly disagree) to 7 (strongly agree). Similar to the relationship between attitudes and behavioural beliefs, the calculation of a normative belief-based measure can be considered in terms of an expectancy-value formula. Fishbein and Ajzen [12], however, concede that this formulation adds little or nothing to the prediction of subjective norms and recommend that descriptive and injunctive normative belief items be combined to provide an index of normative beliefs that determines social norm. Therefore, in this study, descriptive and injunctive normative scores were summed across all the items to create a total normative beliefs score.

##### Perceived Behavioural Control

Direct Measure of PBC

The direct measurement of PBC in this study consisted of three items with an acceptable internal consistency score (α = 0.72). Consistent with Fishbein and Ajzen’s [12] (p. 167) recommendations, both “perceived capacity” and “perceived autonomy” items were included in the questionnaire. Participants were asked to rate each item using a seven-point Likert scale. The mean of the item scores represented an overall PBC score.

Control Beliefs

The modal salient control beliefs identified during the elicitations study (see Table 4) were converted into a set of statements representing the control factors affecting the PA behaviour of pregnant women in East Kent. “Control belief strength” was assessed with five items relating to the control factor’s presence; participants were asked to rate four of the items using a seven-point Likert scale ranging from 1 (strongly disagree) to 7 (strongly agree) and one item ranging from 1 (extremely likely) to 7 (extremely unlikely). For example, “I have adequate knowledge about exercising during pregnancy”.

The “perceived power of the control factor” to influence pregnant women’s PA behaviour was assessed by pregnant women’s agreement with a statement relating to the impact of the identified control factors; participants were asked to rate the five items on a seven-point Likert scale ranging from 1 (strongly disagree) to 7 (strongly agree). For example, “having adequate knowledge about PA during pregnancy will enable me to exercise regularly during my pregnancy”.

Each “control belief” score was multiplied by the corresponding “perceived power to influence behaviour” score and the resulting products summed across all the items to create a total control beliefs score.

## 3. Results

The IBM SPSS (version 22.0) software (Armonk, New York, IBM Corp.) package was used to calculate the statistical findings.

### 3.1. PPAQ

Total activity values (MET hours per week) were calculated with mean values being compared between “time 1” and “time 2” using a paired samples *t*-test (see Table 5). Statistically significant decreases in scores were noted for total activity [*t* (81) = 4.21, *p* < 0.001], sedentary activity [*t* (81) = 2.28, *p* < 0.05], light intensity activity [*t* (81) = 3.39, *p* < 0.01], moderate intensity activity [*t* (81) = 3.15, *p* < 0.01], and occupational activity [*t* (81) = 5.46, *p* < 0.001]. Scores for vigorous activity, household/caregiving activity, and sport and exercise activities did not significantly change between “time 1” and “time 2”.

### 3.2. Theory of Planned Behaviour

The recommended statistical procedure for examining the predictive utility of the TPB is hierarchical regression analysis (HRA) [10]. However, as regression analysis is very sensitive to outliers, an initial regression run was carried out to identify cases poorly fitting the model [54]. Inspection of residuals and Mahalanobis distances identified five potential cases as outliers. Following inspection, four of these were removed and excluded from the subsequent analyses. The remaining sample size (n = 78) had adequate power (based on a power of 0.80 and an alpha of 0.05) to conduct regression analyses with two and three predictor variables (N > 50 + 8 m, where m is the number of independent variables) [55].

### 3.3. Group Differences

Multivariate analysis of variance (MANOVA) was used in the first instance to compare sample characteristics across the TPB constructs (or dependent variables). The data set was screened for normality, linearity, homoscedasticy, and multicollinearity, with no serious violations noted. No group differences were found for the independent categorical variables of marital status, employment status, level of education, pregnancy viability (≥24 weeks, gravidity (number of times a woman has been pregnant), and previous pregnancy complications. There were insufficient data to examine group differences between all three trimesters; however, no group differences were found between trimesters two and three.

There was, however, a statistically significant difference between those who reported participating in regular PA during their pregnancy (*n* = 43) and those who indicated that they did not (*n* = 34), F (5, 71) = 7.87, *p* = 0.000; Wilks’ Lambda = 0.64; (Pillai’s Trace = 0.36); partial eta squared = 0.36. Significant group differences were detected across all of the TPB measures, with women who were regularly physically active scoring higher on all of the measures compared to women who were not [Bonferroni adjustment: *p* < 0.01; behaviour (*p* = 0.000; partial eta squared = 0.31 or 31%), intention (*p* = 0.000; partial eta squared = 0.213 or 21.3%), attitude (*p* = 0.001; partial eta squared = 0.128 or 12.8%), subjective norm (*p* = 0.002; partial eta squared = 0.125 or 12.5%) and PBC (*p* = 0.000; partial eta squared = 0.184 or 18.4%)].

### 3.4. Strength of Relationships

Pearson correlations were next examined to assess the strength of the relationships among the TPB constructs. These product-moment correlation coefficients are presented in Table 6. In terms of the original TPB, intention (r = 0.75; *p* < 0.01) and PBC (r = 0.47; *p* < 0.01) had the strongest associations with PA behaviour. PBC (r = 0.56; *p* <.01) had the strongest correlation with intention; however, both attitude (r = 0.51; *p* = < 0.01) and subjective norm (r = 0.53; *p* < 0.01) were strongly associated with intention.

The concurrent validity of direct measures was assessed by association with their corresponding belief index; behavioural beliefs were significantly correlated with attitude (r = 0.72; *p* < 0.01), normative beliefs were significantly correlated with subjective norm (r = 0.85; *p* < 0.01), and control beliefs were significantly correlated with PBC (r = 0.62; *p* < 0.01). Thus, the beliefs elicited in this study accurately represented the true beliefs of pregnant women in East Kent.

When considering past behaviour during pregnancy as a variable alongside the original TPB variables, past behaviour had a strong association with future PA behaviour (r = 0.79; *p* = < 0.01), intention (r = 0.91; *p* = < 0.01), and PBC (r = 0.60; *p* = < 0.01), and a medium correlation with attitude (r = 0.45; *p* = < 0.01), and subjective norm (r = 0.48; *p* = < 0.01). Although it is acknowledged that high correlations may be an indication of multicollinearity, neither the tolerance values nor variance inflation factors (VIF) in any of the independent variables approached the cut-off points [53]. Furthermore, Tabachnick and Fidell [54] suggest that collinearity can be ignored where the purpose of analysis is prediction or in studies where repeated measures of the same variable occur. In this application of the TPB, past behaviour, intention, and ultimate behaviour were all measured on the same scale to ensure compatibility.

### 3.5. Predictive Utility

The main aim of this study was to examine the predictive utility of the TPB in explaining pregnant women’s PA intentions and behaviour and to scrutinise the role of past behaviour within three scenarios: (1) the original TPB, (2) controlling for the influence of past behaviour, and (3) considering past behaviour as an additional TPB variable.

To examine the hypotheses concerning the predictive utility of the TPB in its original form, two separate forced HRA were performed (see Table 7). The content and order in which the blocks entered were based on the theoretical principles of the TPB [10].

In the first HRA, PA behaviour (dependent variable) was regressed on intention (block 1), followed by PBC (block 2), and attitude and subjective norm (block 3). The results showed that intention (block 1) explained 56.1% of the variance in pregnant women’s PA behaviour, F (1, 76) = 96.98, *p* < 0.001. PBC (block 2) explained an additional 0.3% of the variance, F (2, 75) = 48.49, *p* < 0.001; however, whilst intention was a significant predictor of PA behaviour (β = 0.71, *p* < 0.001), PBC was not (β = 0.07, *p* = 0.46). The addition of attitude and subjective norm (block 3) explained a further 0.6% of the variance in PA behaviour, F (4, 73) = 24.18, *p* < 0.001, with only intention making a unique statistically significant contribution (β = 0.74, *p* < 0.001). This final model indicates that intention uniquely explains 33.1% of the variance in pregnant women’s PA behaviour (part correlation = 0.575).

In the second HRA, intention (dependent variable) was regressed on attitude and subjective norm (block 1), followed by PBC (block 2). Together, attitude and subjective norm (block 1) explained 35.1% of the variance in pregnant women’s intention to be physically active, F (2, 75) = 20.25, *p* < 0.001, with both attitude (β = 0.31, *p* < 0.01) and subjective norm (β = 368, *p* < 0.01) making unique statistically significant contributions. PBC (block 2) explained an additional 5.1% of the variance in pregnant women’s PA intentions, F (3, 74) = 16.54, *p* < 0.001, with subjective norm (β = 0.29, *p* < 0.05) maintaining its unique contribution and PBC (β = 0.31, *p* < 0.05) providing an additional unique contribution. Attitude, however, failed to maintain a unique contribution (β = 0.15, *p* = 0.23). This final model indicates that subjective norm uniquely explained 5.29% of the variance in pregnant women’s PA intention (part correlation = 0.230) whilst PBC indicated a unique contribution of 5.06% (part correlation = 0.225).

To examine the hypotheses concerning the predictive utility of the TPB in the second scenario, two separate HRA were performed to assess the ability of original TPB constructs to predict the PA behaviour and intention of pregnant women, after controlling for the influence of past behaviour (see Table 8). Here, the influence of past behaviour was statistically controlled for by entering it into the first block of the HRA.

In the first HRA, PA behaviour (dependent variable) was regressed on past behaviour (block 1), followed by intention (block 2), and PBC (block 3). Results showed that past behaviour (block 1) explained 62.9% of the variance in pregnant women’s PA behaviour, F (1, 76) = 128.80, *p* < 0.001. After entry of intention (block 2), the total variance explained by the model as a whole was 63.3%, F (2, 75) = 64.66, *p* < 0.001; however, the additional contribution provided by intention was not statistically significant: R squared change = 0.004, F change (1, 75) = 0.82, *p* = 0.37. Following the addition of PBC (block 3), the model as a whole remained significant: F (3, 74) = 42.56, *p* < 0.001; however, PBC did not explain any further variance in PA behaviour: R squared change = 0.000, F change (1, 74) = 0.03, *p* = 0.87.

In the second HRA, intention (dependent variable) was regressed on past behaviour (block 1), followed by attitude and subjective norm (block 2), and PBC (block 3). Past behaviour (block 1) explained 83% of the variance in pregnant women’s intention to be physically active, F (1, 76) = 372.19, *p* < 0.001. After entry of attitude and subjective norm (block 2), the model as a whole explained 84.6% in variance, F (3, 74) = 135.92, *p* < 0.001; however, whilst the additional contribution provided by attitude and subjective norm was significant—R squared change = 0.016, F change (2, 74) = 3.85, *p* < 0.05—neither attitude (β = 0.09, *p* = 13) nor subjective norm (β = 08, *p* = 0.16) made any unique contributions. Following the addition of PBC (block 3), the model as a whole remained significant, F (4, 73) = 103.03, *p* < 0.001; however, PBC did not contribute significantly to the prediction of intention: R squared change = 0.003, F change (1, 73) = 1.52, *p* = 0.22.

To examine the hypotheses concerning the predictive utility of the TPB in the third scenario, two separate HRA were performed (see Table 9). Here, past behaviour was considered as an additional variable and entered in the last block of the HRA.

In the first HRA, PA behaviour (dependent variable) was regressed on intention (block 1), followed by PBC (block 2), and past behaviour (block 3). The results showed that intention (block 1) explained 56.1% of the variance in pregnant women’s PA behaviour: F (1, 76) = 96.98, *p* < 0.001. After entry of PBC (block 2), the model as a whole explained 56.4% of the variance in PA behaviour: F (2, 75) = 48.49, *p* < 0.001; however, the additional contribution provided by PBC was not statistically significant: R squared change = 0.003, F change (1, 75) = 0.56, *p* = 0.46. Only intention provided a unique statistically significant contribution (β = 0.71, *p* < 0.001) to the variance explained by the model. Following the addition of past behaviour (Block 3), the model as a whole explained 63.3% in variance: F (3, 74) = 42.56, *p* < 0.001. This additional contribution added significantly to the prediction of exercise behaviour: R squared change = 0.069, F change (1, 74) = 13.95, *p* < 0.001; however, intention failed to maintain its unique contribution (β = 0.16, *p* = 0.37) and only past behaviour made a unique statistically significant contribution (β = 0.66, *p* < 0.001). This final model indicates that past behaviour uniquely explains 6.92% of the variance in pregnant women’s PA behaviour (part correlation = 0.263).

In the second HRA, intention (dependent variable) was regressed on attitude and subjective norm (block 1), followed by PBC (block 2), and past behaviour (block 3). Together, attitude and subjective norm (block 1) explained 35.1% of the variance in pregnant women’s intention to be physically active—F (2, 75) = 20.25, *p* < 0.001—with both attitude (β = 0.31, *p* < 0.01) and subjective norm (β = 36, *p* < 0.01) making unique statistically significant contributions. After entry of PBC (block 2), the model as a whole explained 40.1% of the variance in PA behaviour: F (3, 74) = 16.54, *p* < 0.001. The additional contribution provided by PBC was statistically significant—R squared change = 0.051, F change (1, 74) = 6.23, *p* < 0.05—with subjective norm (β = 0.29, *p* < 0.05) maintaining its unique contribution and PBC (β = 0.31, *p* < 0.05) providing an additional unique contribution. Attitude, however, failed to maintain a unique contribution (β = 0.15, *p* = 0.23). Following the addition of past behaviour (block 3), the model as a whole explained 85% of the variance in pregnant women’s PA intentions: F (4, 73) = 103.03, *p* < 0.001. The additional contribution of past behaviour added significantly to the prediction of intention—R squared change = 0.448, F change (1, 73) = 217.41, *p* < 0.001—with only past behaviour providing a unique statistically significant contribution (β = 0.86, *p* < 0.001). Neither subjective norm (β = 0.09, *p* = 0.11) nor PBC maintained their respective unique contributions (β = −0.08, *p* = 0.22). This final model indicates that past behaviour uniquely explained 44.8% of the variance in pregnant women’s PA intention (part correlation = 0.669).

## 4. Discussion

The main aim of this study was to examine the predictive utility of the TPB in explaining pregnant women’s PA intentions and behaviour and to scrutinise the role of past behaviour within the context of pregnancy. Specifically, it was hypothesized that: (1) concerning the original TPB, intention will be the strongest determinant of PA behaviour, and attitude will be the strongest determinant of intention; (2) when controlling for past behaviour, the influence of intention and PBC on future behaviour will be attenuated with unique influences remaining, and the influence of attitudes, subjective norms and PBC on intentions will be attenuated with unique influences remaining; and (3) when adding past behaviour as additional variable, the model will explain significantly more variance in pregnant women’s PA intentions and behaviour.

### 4.1. The Original TPB

When considering the original TPB as a conceptual framework, intention emerged as the strongest determinant of pregnant women’s PA behaviour. Together, intention and PBC explained 56.4% of the variance in pregnant women’s PA behaviour; however, only intention emerged as a significant predictor. This finding reflects that of Symons Downs and Hausenblas [24] regarding PA behaviour in general and Symons Downs and Hausenblas [50,52] who reported intention to be physically active a stronger predictor of behaviour than perception of control. It could therefore be argued that the influence of PBC on pregnant women’s PA behaviour may not be particularly realistic. Ajzen [57] advises that the association between PBC and behaviour will only transpire when an individual’s perception of control matches their actual control over the behaviour of interest. Pregnant women may thus be faced with uncertainty regarding the factors that could enable or prevent them from initiating or continuing with regular physical activities.

The perceived ease or difficulty with which pregnant women can engage in physical activities emerged as an important determinant of their motivation (or willingness) to participate in regular PA. This finding reflects that of Black, Kieffer, Villarruel, and Sinco [58] (p. 8), who showed that the “ability to overcome environmental barriers” and “ability to overcome personal barriers” predicted the PA intention of pregnant Latina women (N = 98). Consequently, to understand why pregnant women hold certain perceptions of control and to ultimately produce a change in PBC, it is necessary to consider the cognitive foundations underlying this determinant [57]. In this study, control beliefs were strongly correlated with the direct measure of PBC, thereby suggesting that pregnant women’s perception of their capacity to participate in regular physical activities were accurately represented. Thus, redressing some of these control beliefs or making available new beliefs may lead to changes in pregnant women’s perceptions of autonomy and control [57].

Whilst PBC did not directly influence PA behaviour in this study, it did play an important role in the formation of pregnant women’s intention to be physically active. Together, the three determinants of intention explained 40.2% of the variance in pregnant women’s PA motivation, with subjective norm and PBC providing unique contributions. Contrary to the hypothesis, attitude failed to provide a unique contribution and subjective norm emerged as marginally stronger in predicting intention than PBC. This finding is similar to Symons Downs and Hausenblas [52], who reported subjective norm as the strongest predictor of PA intention during the third trimester and reflects the findings of Hausenblas and Symons Downs [11], who report subjective norm as a significant predictor of PA intention during the first trimester of pregnancy. Furthermore, Symons Downs and Hausenblas [50] note that in their study of second trimester PA intentions and behaviour, attitude was only marginally stronger in predicting intention than PBC.

The fact that subjective norm predicted pregnant women’s PA intentions contrasts with the meta-analysis of Symons Downs and Hausenblas [24], who reported that subjective norm did not predict PA intentions in the PA domain. However, it should be noted that the present study included measures of injunctive and descriptive norms, which has not always been the case with studies involving the TPB. In his meta-analysis concerning the effect of subjective norms on behaviour in the TPB, Manning “supports the distinction between descriptive norms and injunctive norms and underscores the recommendation to include both types of norms in planned behaviour research” [59] (p. 687). Future studies investigating pregnant women’s PA intentions should therefore also aim to include a higher-order construct that represents both injunctive and descriptive norms.

Reflecting the findings of De Vivo et al. [39], the present study showed that pregnant women’s perception of the social pressure to participate in regular PA is a key factor in determining their motivation to engage with the behaviour. In accordance with TPB principles, any intervention that is aimed at influencing social norms should address the corresponding salient normative beliefs as they provide the cognitive foundation on which the determinant is based [57]. Thus, to assess whether elicited beliefs accurately represent the perceptions of the population under investigation, it is necessary for normative beliefs to correlate with the direct measure of subjective norm [12]. The results of the present study confirm a strong relationship between normative beliefs and subjective norm. Addressing inconsistent perceptions held by healthcare professionals (e.g., midwives and health visitors) and the wider family network may have a positive effect on pregnant women’s view of social support to regularly engage in PA.

### 4.2. Controlling for the Influence of Past Behaviour

Controlling for past behaviour attenuated the influence of intention and PBC on behaviour, with neither of the original variables providing a unique influence. Thus, PA behaviour within the stable context of pregnancy predicted PA behaviour later in the pregnancy. The current findings echo those of Zamora-Flyr [40] who defined PA behaviour as walking and found that within the original TPB, intention was the only independent predictor of walking behaviour later on in pregnancy. However, the addition of past walking behaviour extinguished the effects of intention and past behaviour emerged as the only predictor of future walking behaviour. Controlling for past behaviour in this study also attenuated the influence of attitude, subjective norms and PBC on intention, with none of the original variables providing a unique influence. Thus, contrary to the hypothesis, this finding supports a direct relationship between past behaviour and intention.

### 4.3. Past Behaviour as an Additional Variable

Thirdly, consistent with the hypothesis, the addition of past behaviour led to an increase in the predictive utility of the TPB. Together, intention, PBC and past behaviour explained 63.3% of the variance in pregnant women’s PA behaviour, with only past behaviour making a unique statistically significant contribution. When combined, attitude, subjective norm, PBC and past behaviour explained 85% of the variance in pregnant women’s PA intention, with only past behaviour providing a unique statistically significant contribution. However, given the fact that past behaviour also had a strong relationship with PBC and a medium relationship with attitude and subjective norm, it is not unreasonable to suggest that these constructs also act indirectly in predicting pregnant women’s PA intentions. Indeed, Yordy and Lent [60] suggest that TPB “variables may serve as partial link between past behaviour and future PA participation” (p. 371).

Contrasting McEachan et al.’s [37] findings in the health domain, where past behaviour was the most important predictor of behaviour but not intention, the influence of past behaviour in this study was most notable in the prediction of pregnant women’s PA intentions, uniquely explaining 45% of the variance observed. Thus, it could be argued that regularly engaging in PA during pregnancy reduces cognitive deliberation over whether to remain active as pregnancy progresses. Active pregnant women may simply be adapting existing routines rather than deliberating whether they should be active. Furthermore, Yordy and Lent [60] suggest that regular exercisers may previously have developed strategies for dealing with common barriers. For example, pregnant women may already have childcare arrangements in place, which allows them to continue engagement with physical activities. It is also possible that pregnant women who were already participating in a specific type or intensity of PA (e.g., walking) may have continued to do so without the need for any contemplation, which could suggest that being active is a valued part of a healthy lifestyle.

Similarly to Symons Downs and Hausenblas [52], the influence of past behaviour was also observed when group differences between active and inactive women were examined. Pregnant women who classed themselves as active scored significantly higher across all of the TPB measurements, with the greatest differences noted in intention (21.3%) and future behaviour (31%). This is an important finding as it suggests behaviour status as a moderator of pregnant women’s PA intentions and behaviour. This also supports Currie et al.’s [42] notion that PA at baseline is an important consideration as it has the potential to influence the outcome of an intervention to a greater extent than the intervention itself. 

**Figure 2:**
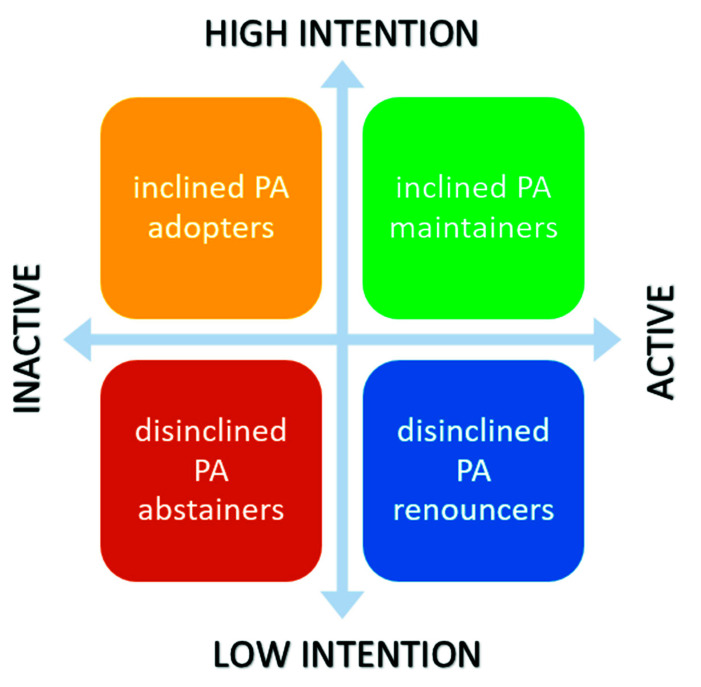
Pregnancy physical activity profiling.

Intention (or an individual’s stated orientation towards behaviour) represents the motivational factors of (1) attitude (a construct based on behavioural beliefs around the likely consequences of engaging in a specific behaviour); (2) subjective norm (a construct based on normative beliefs representing the perceived pressure to conform to the perceptions of significant others regarding a specific behaviour); and (3) PBC (a construct based on control beliefs signifying the perceived ability with which one can carry out a specific behaviour); [12,13]. Whilst McEachan et al. [37] suggest that past behaviour tendencies are not as easily changed as these motivational factors and that such findings are of concern when devising behavioural interventions, the outcome of this current study presents healthcare professionals and researchers with an opportunity to introduce tailored advice and interventions based on the profiling of pregnant women. Specifically, four profile types are being proposed to match intention and behaviour status (see Figure 2): (1) women who have been regularly active in the past and intend to continue being physically active throughout their pregnancy, i.e., *inclined PA maintainers*; (2) pregnant women who have been active in the past and do not intend to maintain their PA routine, i.e., *disinclined PA renouncers*; (3) women who have been inactive but intend to be physically active during their pregnancy, i.e., *inclined PA adopters*; and (4) pregnant women who have been inactive in the past and do not intend to be physically active during their pregnancy, i.e., *disinclined PA abstainers*. Future research should explore these proposed scenarios and investigate which techniques and strategies may be best-suited to each. For example, strategies such as very brief advice [61] may be more suited to inclined PA maintainers; a person-centered approach involving goal setting may be required to re-engage disinclined PA renouncers; motivational interviewing (MI) could be utilised to support inclined PA adopters, whereas a PA counselling approach (incorporating behaviour change and MI techniques) may be required to engage disinclined PA abstainers.

## 5. Limitations

The PPAQ, a self-reported and self-administered questionnaire, was used to measure the type, duration, frequency, and intensity of total activity in pregnant women. However, it was observed that pregnant women in this sample grossly overestimated their activity levels to the extent that their accounts were considered unrealistic (i.e., there were not enough hours in the day to account for the duration of activities as reported by some participants). It is possible that some pregnant women may have been concerned about the way they would be perceived or thought that they had to portray a certain image (i.e., self-presentation bias; [12]). Nonetheless, it is recognised that the PPAQ is not primarily concerned with the absolute measurement of energy expenditure but rather with ranking individuals with respect to their PA levels [52]. It should also be noted that measures obtained by the PPAQ were not used in any analyses pertaining to the predictive utility of the TPB. Instead, the behavioural criterion measure was used (see [12,43,44]). Future studies may wish to consider the objective measurement PA behaviour, although this is also not without significant challenges in a pregnant sample [62]. Further research should consider improvements to measures of PA during pregnancy; specifically, research should aim to (a) clarify the exercise-dose response relationship associated with health benefits during pregnancy; (b) establish the MET intensities associated with various physical activities during pregnancy; and (c) develop and investigate novel measures that are both cost-effective and reliable, e.g., non-exercise activity thermogenesis (NEAT) [63,64].

A further limitation to note was that there was not adequate power to split and analyse the data based on the dichotomous criterion of behavioural status (i.e., whether or not participants were meeting the guidelines for moderate intensity physical activity). Although not the aim of this study, it may have been useful to determine the predictive utility of the TPB in terms of those who reported that they were exercising regularly (*n* = 43) and those who reported that they were not (*n* = 34). In particular, it is recognised that the TPB variables may be more important in this adoption phase as opposed to the maintenance of the behaviour [8]. A split sample would have, however, resulted in inadequate power and therefore also the potential of error. The role of past behaviour was instead considered in terms of the reported frequency of past behaviour (frequency criterion).

## 6. Conclusions

Pregnancy is a life event that requires conscious re-evaluation of existing beliefs, values, and strategies. For women who were active before becoming pregnant, this process may be less consuming; however, for those who were not previously active, more support may be required in terms of addressing concerns, education, overcoming barriers, and offering reassurance. Pregnancy PA profiling based on intention and behaviour status is introduced here as a novel yet practical framework which presents healthcare professionals with the opportunity and structure to provide tailored advice and guidance to pregnant women, thereby facilitating engagement with PA throughout motherhood. This framework also offers a unique vantage point from which further interventions and research can follow.

## Figures and Tables

**Table 1:** Modal salient behavioural beliefs of pregnant women in East Kent.

**Advantages**	***N* = 35**	**Percentage**	**Cumulative Percentage**
Improved physical fitness	13	24.53	24.53
Improved general health	12	22.64	47.17
Weight management	6	11.32	58.49
Better prepared for labour	5	9.43	67.92
Psychological wellbeing	5	9.43	77.35
**Disadvantages**	***N* = 28**	**Percentage**	**Cumulative Percentage**
Fatigue	9	32.14	32.14
Overdoing it	5	17.86	50.00
Fear of harming baby	5	17.86	67.86
Injury	3	10.71	78.57

**Table 2:** Modal salient injunctive normative beliefs of pregnant women in East Kent.

**Approve**	***N* = 40**	**Percentage**	**Cumulative Percentage**
Health professionals	15	37.50	37.50
Family	8	20.00	57.50
Friends	6	15.00	72.50
Husband/partner	4	10.00	82.50
Exercise professionals	4	10.00	92.50
**Disapprove**	***N* = 18**	**Percentage**	**Cumulative Percentage**
Family	4	22.22	22.22
Health professionals	3	16.67	38.89
Friends	2	11.11	50.00
Society in general	2	11.11	61.11
Older people	2	11.11	72.22
Complicated pregnancies	2	11.11	83.33

**Table 3:** Modal salient descriptive normative referents of pregnant women in East Kent.

**Most likely**	***N* = 28**	**Percentage**	**Cumulative Percentage**
Active people	14	50.00	50.00
Health/exercise professionals	4	14.29	64.29
Experienced mums	2	7.14	71.43
Those without health issues	2	7.14	78.57
Those without dependents	2	7.14	85.71
**Least likely**	***N* = 32**	**Percentage**	**Cumulative Percentage**
Those with health issues	10	31.25	31.25
Inactive people	6	18.75	50.00
Those who suffered previous loss	6	18.75	68.75
First pregnancy	4	12.50	81.25

**Table 4:** Modal salient control beliefs of pregnant women in East Kent.

**Easy/Enable**	***N* = 35**	**Percentage**	**Cumulative Percentage**
Increased access and availability	9	25.71	25.71
Having more time available	7	20.00	45.71
Improved knowledge	5	14.29	60.00
Affordability	5	14.29	74.29
Suitable activity structure	5	14.29	88.58
**Difficult/prevent**	***N* = 41**	**Percentage**	**Cumulative Percentage**
Health issues	11	26.83	26.83
Not having enough time	10	24.39	51.22
Fatigue	6	14.63	65.85
Having dependents	4	9.76	75.61
Limited access	4	9.76	85.37

**Table 5:** Paired samples *t*-test representing PPAQ difference in MET scores between time 1 and time 2 (n = 82).

Variable	Time	Mean (MET Hours Per Week)	SD	*t*	Sig. (2-Tailed)
Total activity	1	301.28	123.76	4.21	0.000
	2	252.25	113.10		
Sedentary behaviour	1	68.32	31.18	2.28	0.026
	2	61.14	30.48		
Light intensity activity	1	119.50	57.44	3.39	0.001
	2	104.94	50.83		
Moderate intensity activity	1	110.94	93.91	3.15	0.002
	2	83.54	75.53		
Vigorous activity	1	2.43	4.82	−0.51	0.614
	2	2.64	5.08		
Household/caregiving activity	1	117.88	77.48	1.52	0.132
	2	110.82	72.65		
Occupational activity	1	96.11	89.31	5.47	0.000
	2	53.73	60.68		
Sport and exercise activity	1	13.30	10.53	−0.10	0.922
	2	13.39	11.02		

**Table 6:** Pearson correlations (r) between variables.

*N* = 78	Behaviour	Intention	Attitude	Subjective Norm	PBC	Past Behaviour	Behavioural Beliefs	Normative Beliefs	Control Beliefs
Behaviour									
Intention	0.75 **								
Attitude	0.40 **	0.51 **							
Subjective Norm	0.35 **	0.53 **	0.55 **						
PBC	0.47 **	0.56 **	0.65 **	0.53 **					
Past Behaviour	0.79 **	0.91 **	0.45 **	0.48 **	0.60 **				
Behavioural Beliefs	0.17	0.30 **	0.72 **	0.53 **	0.45 **	0.25 *			
Normative Beliefs	0.38 **	0.55 **	0.65 **	0.85 **	0.59 **	0.51 **	0.57 **		
Control Beliefs	0.27 *	0.45 **	0.64 **	0.56 **	0.62 **	0.44 **	0.56 **	0.62 **	

** Correlation is significant at the 0.01 level (two-tailed). * Correlation is significant at the 0.05 level (two-tailed). (Small r = 0.10 to 0.29; medium r = 0.30 to 0.49; large r = 0.50 to 1.0) [56].

**Table 7:** HRA for the original TPB.

Variable	*R* ^2^	F	Δ*R*^2^	ΔF	Beta	Part *r*
**Predicting behaviour**						
Block 1:	0.561	96.981 **				
1. Intention					0.749 **	
Block 2:	0.564	48.491 **	0.003	0.561		
1. Intention					0.710 **	0.590
2. PBC					0.069	0.057
Block 3:	0.570	24.184 **	0.006	0.510		
1. Intention					0.743 **	0.575
2. PBC					0.098	0.069
3. Attitude					0.007	0.005
4. Subjective Norm					−0.099	−0.076
**Predicting intention**						
Block 1:	0.351	20.250 **				
1. Attitude					0.309 *	0.259
2. Subjective Norm					0.363 *	0.303
Block 2:	0.401	16.536 **	0.051	0.6.266 *		
1. Attitude					0.152	0.110
2. Subjective Norm					0.286 *	0.230
3. PBC					0.308 *	0.225

** Significant at the 0.001 level. * Significant at the 0.05 level.

**Table 8:** HRA for TPB when controlling for past behaviour.

Variable	*R* ^2^	F	Δ*R*^2^	ΔF	Beta	Part *r*
**Predicting behaviour**						
Block 1:	0.629	128.80 **				
1. Past behaviour					0.793 **	
Block 2:	0.633	64.66 **	0.004	0.820		
1. Past behaviour					0.653 **	0.269
2. Intention					0.154	0.063
Block 3:	0.633	42.56 **	0.000	0.028		
1. Past behaviour					0.660 **	0.263
2. Intention					0.155	0.064
3. PBC					−0.015	−0.012
**Predicting intention**						
Block 1:	0.830	372.19 **				
1. Past behaviour					0.911 **	
Block 2:	0.846	135.92 **	0.016	3.85 *		
1. Past behaviour					0.832 **	0.704
2. Attitude					0.087	0.070
3. Subjective norm					0.083	0.066
Block 3:	0.850	103.03 **	0.003	1.52		
1. Past behaviour					0.860 **	0.669
2. Attitude					0.122	0.088
3. Subjective Norm					0.094	0.073
4. PBC					−0.083	−0.056

** Significant at the 0.001 level. * Significant at the 0.05 level.

**Table 9:** HRA for TPB with past behaviour as an additional variable.

Variable	*R* ^2^	F	Δ*R*^2^	ΔF	Beta	Part *r*
**Predicting behaviour**						
Block 1:	0.561	96.981 **				
1. Intention					0.749 **	
Block 2:	0.564	48.491 **	0.003	0.561		
1. Intention					0.710 **	0.590
2. PBC					0.069	0.057
Block 3:	0.633	42.556 **	0.069	13.946 **		
1. Intention					0.155	0.064
2. PBC					−0.015	−0.012
3. Past behaviour					0.660 **	0.263
**Predicting intention**						
Block 1:	0.351	20.250 **				
1. Attitude					0.309 *	0.259
2. Subjective Norm					0.363 *	0.303
Block 2:	0.401	16.536 **	0.051	0.6.266 *		
1. Attitude					0.152	0.110
2. Subjective Norm					0.286 *	0.230
3. PBC					0.308 *	0.225
Block 3:	0.850	103.026 **	0.448	217.41 **		
1. Attitude					0.122	0.088
2. Subjective Norm					0.094	0.073
3. PBC					−0.083	−0.056
4. Past behaviour					0.860 **	0.669

** Significant at the 0.001 level. * Significant at the 0.05 level.

## Data Availability

Further information concerning the mixed methods multiphase research project and additional data pertaining to this transcript is available in the CCCU Research Space Repository, https://bit.ly/3p2OCom.
